# The effect of budesonide/formoterol maintenance and reliever therapy on the risk of severe asthma exacerbations following episodes of high reliever use: an exploratory analysis of two randomised, controlled studies with comparisons to standard therapy

**DOI:** 10.1186/1465-9921-13-59

**Published:** 2012-07-20

**Authors:** Roland Buhl, Piotr Kuna, Matthew J Peters, Tomas LG Andersson, Ian P Naya, Stefan Peterson, Klaus F Rabe

**Affiliations:** 1 Mainz University Hospital, Langenbeckstrasse 1, Mainz, D-55131, Germany; 2 Medical University of Lodz, Lodz, Poland; 3 Concord Hospital, Concord, Australia; 4 Former employee of AstraZeneca R&D, Lund, Sweden; 5 Department of Medicine and Clinic Grosshansdorf, University Kiel, Kiel, Germany

**Keywords:** Asthma, Asthma in primary care

## Abstract

**Background:**

Divergent strategies have emerged for the management of severe asthma. One strategy utilises high and fixed doses of maintenance treatment, usually inhaled corticosteroid/long-acting β_2_-agonist (ICS/LABA), supplemented by a short-acting β_2_-agonist (SABA) as needed. Alternatively, budesonide/formoterol is used as both maintenance and reliever therapy. The latter is superior to fixed-dose treatment in reducing severe exacerbations while achieving similar or better asthma control in other regards. Exacerbations may be reduced by the use of budesonide/formoterol as reliever medication during periods of unstable asthma. We examined the risk of a severe exacerbation in the period after a single day with high reliever use.

**Methods:**

Episodes of high reliever use were quantified and exacerbations occurring post-index day with these episodes were examined *post hoc* in two double-blind studies comparing the efficacy and safety of budesonide/formoterol maintenance and reliever therapy (Symbicort SMART™, Turbuhaler®) 160/4.5 μg twice daily plus as needed with similar or higher maintenance doses of ICS/LABA plus SABA or formoterol.

**Results:**

Budesonide/formoterol maintenance and reliever therapy significantly reduced the risk of episodes of high reliever use (>6 inhalations/day) vs. all alternative ICS/LABA regimens. With conventional fixed-dose treatment the need for exacerbation treatment within 21 days ranged from 6.0–10.1% of days post-index for all regimens compared with 2.5–3.4% of days with budesonide/formoterol maintenance and reliever therapy.

**Conclusions:**

Budesonide/formoterol maintenance and reliever therapy reduces the incidence of high reliever episodes and the exacerbation burden immediately following these episodes vs. alternative ICS/LABA plus SABA regimens at up to double the maintenance dose of ICS.

**Trial registration:**

These studies do not have registration numbers as they were conducted before clinical trial registration was required

## Background

Short-acting β_2_-agonists (SABAs) are the mainstay of asthma reliever medication, but patients who over-rely on SABAs have an increased risk of fatal or near-fatal asthma [1-3]. In contrast, inhaled corticosteroids (ICS) used as maintenance therapy reduce this risk [1,2,4]. Regular use of long-acting β_2_-agonists (LABA) is established as the preferred second-line maintenance therapy in combination with ICS. The beneficial effects of ICS/LABA on exacerbation rates, lung function, SABA use and daily symptoms compared with ICS alone are well documented [5]. However, even in patients using ICS/LABA, escalating SABA use remains an indicator of the potential for a severe exacerbation [6,7].

Budesonide/formoterol as maintenance and reliever therapy is well established and incorporated in guidelines [8]. Without compromising any measure of asthma control, compared with other higher fixed-dose steroid-containing regimens, its great attractiveness is in reducing severe exacerbations [9-15].The mechanism for this effect is currently unknown [9,10,15-17].

One explanation is that events initiating unstable asthma, and potentially an exacerbation, occur less frequently or are promptly aborted and thus not recognisably different from the usual asthma state. Alternatively, the exacerbation sequence may commence, but, in some instances, the additional as-needed budesonide/formoterol modifies the course of the event to prevent an exacerbation. The ‘window of opportunity’ represents the period during which symptom-driven increases in reliever and ICS use may act [18].

Study patients were symptomatic, using reliever medication on most days in the run-in period. If an exacerbation were to develop in this population, further increases in reliever use should be seen. The utility of high reliever use events as a marker for an impending exacerbation could be tested by examining the short-term increase in the conversion to a severe exacerbation. A preferred management strategy would reduce the frequency of periods with high reliever use or lower the conversion rate into a severe exacerbation.

We examined episodes of high reliever use and the clinical course in the subsequent 21 days in two large studies. These studies compared the efficacy and safety of budesonide/formoterol maintenance and reliever therapy (160/4.5 μg twice daily [bid] plus as needed) with:

the same maintenance dose of ICS/LABA, budesonide/formoterol, with terbutaline or formoterol as reliever medication and

with budesonide/formoterol or salmeterol/fluticasone at a higher maintenance dose with terbutaline as needed [10, 12].

## Methods

Patients came from two double-blind, parallel-group exacerbation prevention studies (SD-039-0734; Study A [12]; SD-039-0735; Study B [10]). Severe exacerbations were defined as deteriorations in asthma control resulting in hospitalisation, emergency room (ER) treatment or the need for oral corticosteroids for ≥3 days.

Patients were aged ≥12 years with an asthma diagnosis for ≥6 months and ≥1 exacerbation in the last year. All patients used ICS for ≥3 months prior to enrolment and had a forced expiratory volume in 1 second (FEV_1_) ≥50% of predicted normal.

In Study A, [12] patients received budesonide/formoterol 160/4.5 μg (Symbicort® Turbuhaler®, AstraZeneca, Lund, Sweden) bid during a 2-week run-in and throughout the 12-month randomised treatment period. Terbutaline (Bricanyl®, AstraZeneca, Lund, Sweden; 0.4 mg/inhalation) was used as needed during run-in and either formoterol (Oxis®, AstraZeneca, Lund, Sweden; 4.5 μg/inhalation), terbutaline (0.4 mg/inhalation) or budesonide/formoterol (160/4.5 μg/inhalation) as needed during the treatment period ( 1).

**Figure 1:**
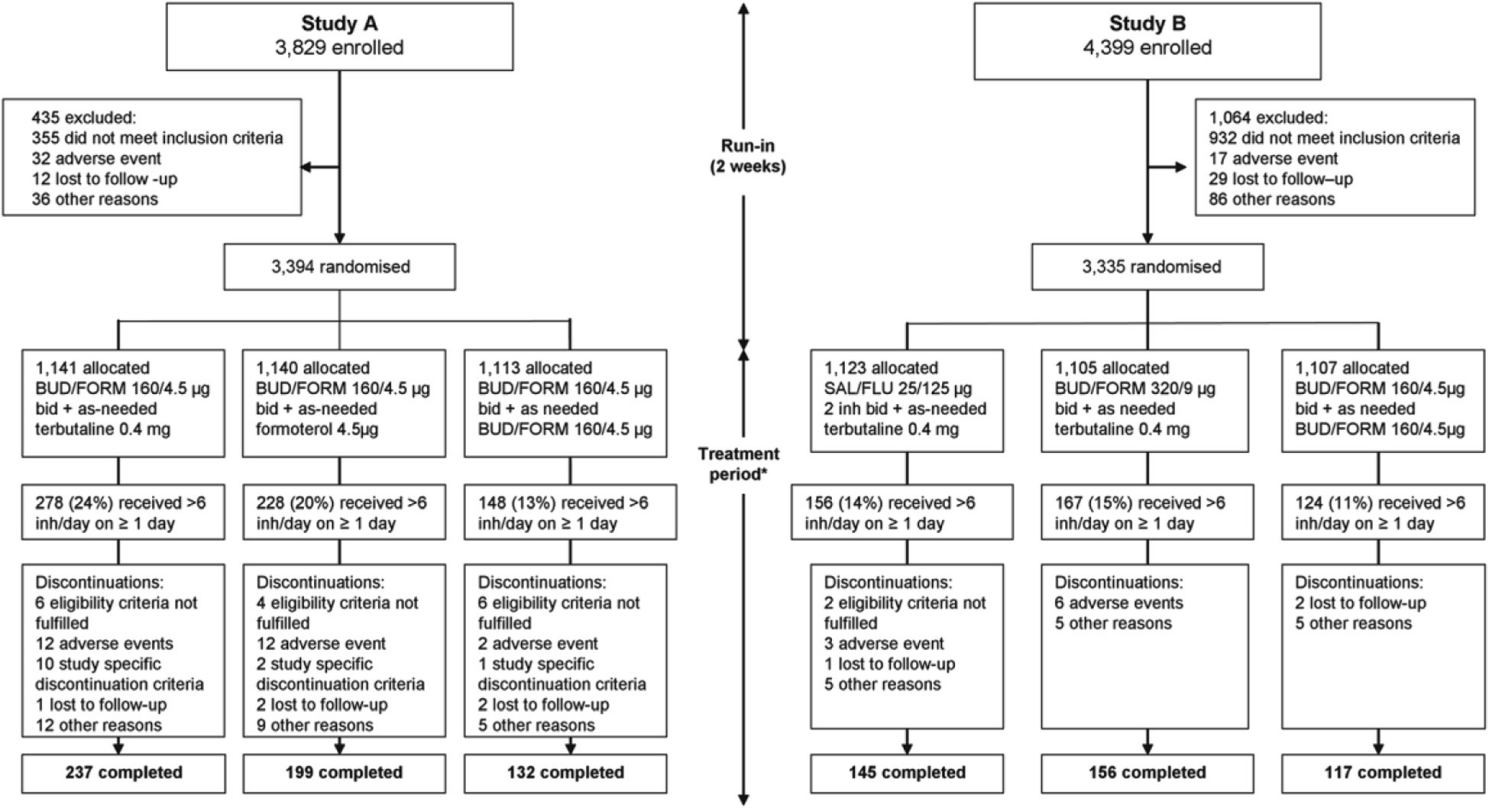
**Patient flow.** *Study A, treatment period = 12 months; Study B, treatment period = 6 months; bid = twice daily; BUD/FORM = budesonide/formoterol; inh = inhalation; SAL/FLU = salmeterol/fluticasone.

In Study B, [10] following a 2-week run-in on ICS plus terbutaline without LABA, patients were randomised to one of three 6-month regimens: budesonide/formoterol (160/4.5 μg bid) for maintenance and reliever as needed, fixed-dose budesonide/formoterol 320/9 μg one inhalation bid plus terbutaline (0.4 mg/inhalation) as needed or fixed-dose salmeterol/fluticasone 25/125 μg (Seretide™ Evohaler™, GlaxoSmithKline, Uxbridge, UK) two inhalations bid plus terbutaline (0.4 mg/inhalation) as needed (Figure 1).

### Increasing SABA use and exacerbations

Analyses focused on confirming the relationship between the first (index) day with a defined threshold of SABA exposure (>2, >4, >6 and >8 inhalations of terbutaline/day for at least one day) and the increased exacerbation risk in subsequent days [10,12].

### Episodes of >6 inhalations/day of reliever

A pre-specified threshold for high reliever use was examined for each regimen. This threshold was selected because maintenance therapy with budesonide/formoterol is approved up to a dose of 1,280/36 μg/day [10,12]. On days when >6 inhalations/day of as-needed therapy were used this limit would be exceeded. We determined the use of >6 as-needed inhalations/day in the intention-to-treat (ITT) populations and the time to the first (index) day with such episodes.

### Exacerbations with >6 inhalations/day of reliever

Exacerbation rates post-index day and the number of subsequent exacerbation days were assessed after the index to study end and post-index for ≤21 days. The 21-day window would allow severe exacerbations coinciding with high reliever use to be captured in reasonable proximity to the index day. The average time in an exacerbation state was defined as the number of days with oral steroid use initiated by the physician or days of hospital treatment, without double-counting if this overlapped. Differences in the annualised exacerbation rate and hospitalisations/ER treatments in these periods were compared between regimens and with reference to analyses in the ITT populations [10,12].

### Tolerability

Descriptive statistics examined patients with an index day of >6 inhalations/day of reliever, to assess the incidence of serious adverse events (SAEs) and discontinuations due to adverse events (DAEs).

### Statistical analysis

The risk profile for patients requiring >6 inhalations/day of reliever in the ITT population was described by Kaplan–Meier plots and evaluated using a Cox proportional hazards model with treatment as factor. All analyses comparing exacerbation rates in the ITT population and post-index rate ratios used a Poisson regression model with treatment as factor and study or post-index time as offset, with confidence intervals adjusted for overdispersion. As data on the percentage of exacerbation days were skewed, *post-hoc* analyses to compare this outcome between treatment regimens were conducted using a bootstrap procedure. This procedure was selected because it does not make assumptions about different data distributions and provides a more controlled estimate of variance.

## Results

### SABA use and exacerbations

**Figure 2:**
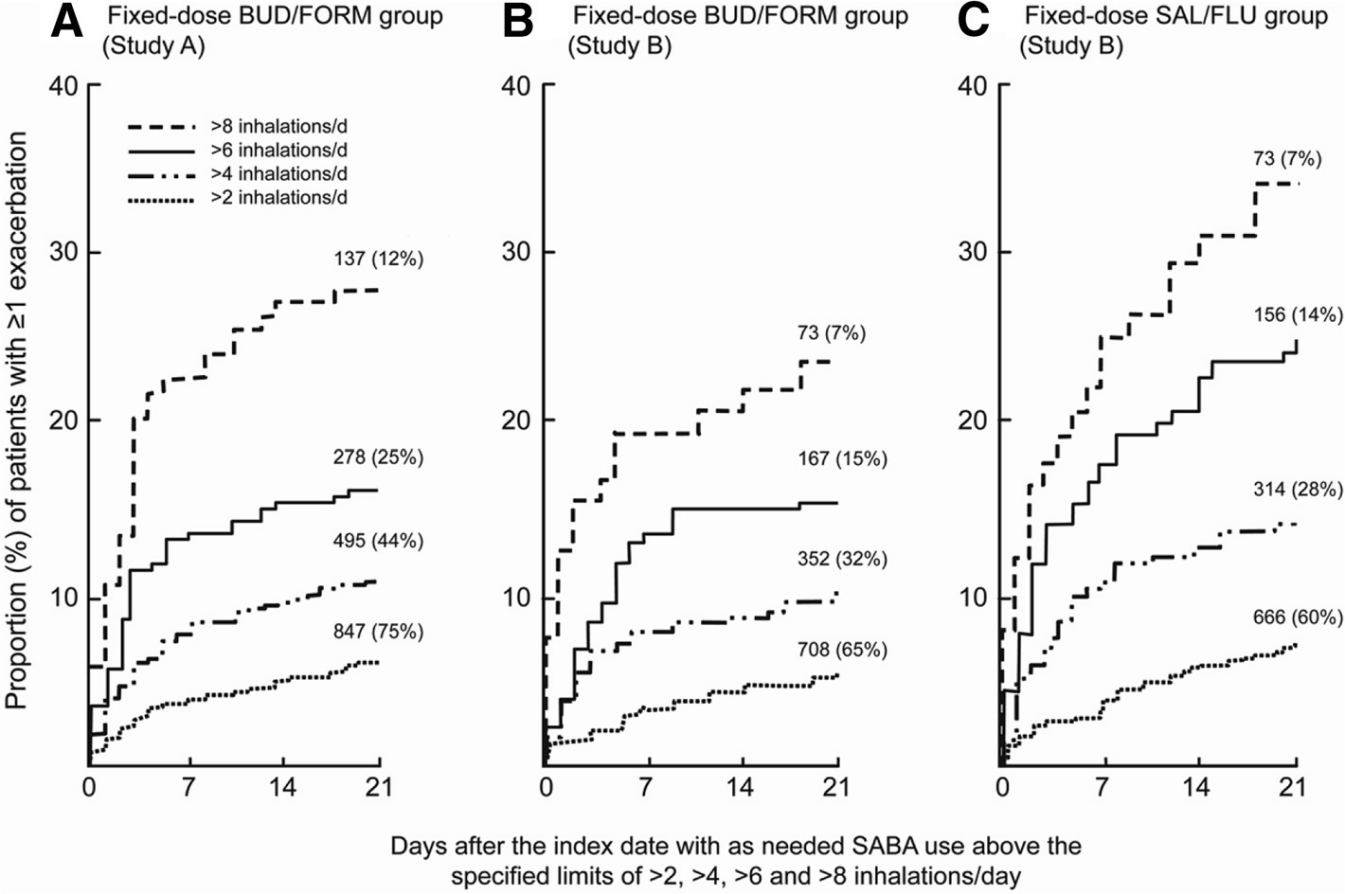
**Incidence of asthma exacerbations occurring 21 days after the first episode with high terbutaline use.** Kaplan–Meier plots of time from first use of >2, >4, >6 and >8 as-needed inhalations/day of terbutaline to first exacerbation on alternative fixed-dose ICS/LABA regimens: A) budesonide/formoterol (BUD/FORM) 160/4.5 μg twice-daily over 12-months (Study A) (N=1,138); B) budesonide/formoterol 320/9 μg twice-daily over 6-months (Study B) (N=1,099); C) salmeterol/fluticasone (SAL/FLU) 50/250 μg twice-daily over 6-months (Study B) (N=1,119). Day 0 is the first day with the specified level of as-needed inhalations of terbutaline. The number of patients with exposure to each level of terbutaline and the percentage of the total randomised population this represents is shown in parenthesis above each Kaplan–Meier curve.

Sensitivity analyses showed that regardless of the ICS/LABA regimen, the higher the number of as-needed inhalations (>2, >4, >6 and >8 inhalations/day) of terbutaline on the index day, the greater the incidence of exacerbations during the following 21 days. Across both studies ~15% of patients using fixed-dose maintenance therapy with budesonide/formoterol (160/4.5 or 320/9 μg bid) and ~24% using salmeterol/fluticasone (50/250 μg bid) experienced severe exacerbations in the 21 days following an index day with >6 inhalations/day of terbutaline (Figure 2).

### >6 inhalations/day of reliever

Patients who used >6 inhalations/day on at least one occasion had a similar baseline demography across treatment regimens (Table 1) and were similar to the ITT populations, although baseline daily reliever use was higher in this subgroup (Additional file 1: Table S1, online supplement).

**Table 1:** Demographics and asthma control during run-in among the patient subgroups with episodes of >6 inhalations/day of as-needed medication use on at least 1 day in Study A and Study B

	Study A (12-month assessment)*			**Study B (6-month assessment)**^**†**^		
	**All patients used BUD/FORM maintenance**			**SAL/FLU FD**	**BUD/FORM FD**	**BUD/FORM maintenance**
	**Terbutaline as needed (n = 278)**	**Formoterol as needed (n = 228)**	**BUD/FORM as needed (n = 148)**	**Terbutaline as needed (n = 156)**	**Terbutaline as needed (n = 167)**	**BUD/FORM as needed (n = 124)**
Male, n (%)	108 (39)	95 (42)	58 (39)	67 (43)	66 (40)	57 (46)
Age, years	43 (12–78)	42 (12–81)	42 (12–77)	38 (12–73)	41 (12–81)	39 (12–74)
Smokers, n (%)	20 (7)	18 (8)	17 (12)	13 (8)	23 (13)	11 (8)
ICS at entry, μg·day^-1^	777 (250–1,600)	789 (400–1,600)	755 (400–1,600)	811 (500–1,600)	796 (300–2,000)	791 (500–1,600)
FEV_1_, % predicted	70 (39–100)	70 (50–99)	71 (50–99)	71 (51–132)	70 (50–117)	72 (50–114)
Reversibility, % (range)	23 (11–90)	24 (12–81)	24 (12–132)	23 (12–79)	23 (11–84)	22 (12–74)
** *Control assessed in run-in* **						
Morning PEF, l/min	325 (111–619)	334 (106–689)	328 (123–621)	324 (127–885)	326 (113–607)	337 (155–558)
Reliever use, inh./day	2.8 (0.5–9.7)	2.9 (0.5–9.1)	3.1 (0.6–8.9)	3.6 (0.7–10.8)	3.7 (0.8–9.5)	3.8 (0.8–8.8)
Symptom-free days, %	7 (0–78)	7 (0–60)	6 (0–100)	6 (0–90)	4 (0–80)	5 (0–60)

*Patients used BUD/FORM maintenance plus terbutaline as needed during run-in.

^†^Patients used pre-study ICS + terbutaline during run-in (no LABA).

Data are presented as mean (range) unless otherwise stated. BUD/FORM = budesonide/formoterol; FD = fixed-dose maintenance; FEV_1_ = forced expiratory volume in 1 second; inh. = inhalation; ICS = inhaled corticosteroids; LABA = long-acting β_2_-agonist; PEF = peak expiratory flow; SAL/FLU = salmeterol/fluticasone.

In the 12-month Study A, an index day of >6 inhalations/day was observed in 278 (24%) of patients on terbutaline, 228 (20%) of patients on formoterol and 148 (13%) of patients on budesonide/formoterol reliever therapy respectively (Figure 1). In the 6-month Study B, an index day of >6 inhalations/day of reliever was observed in 156 (14%) patients on salmeterol/fluticasone, 167 (15%) on budesonide/formoterol and 124 (11%) patients on budesonide/formoterol maintenance and reliever treatment, respectively (Figure 1). In both studies the risk of any episode with >6 inhalations/day of reliever was reduced with budesonide/formoterol maintenance and reliever therapy vs. comparators (*P* ≤ 0.014; Table 2 and 3).

**Table 2:** Annualised exacerbation rates and hospitalisations/ER treatments in the full ITT population and the subgroups with episodes of high as-needed reliever use (>6 inhalations/day on ≥1 study day) (Study A)

	**Reliever treatment groups**			**Risk or rate ratios (95% CI), comparing reliever groups**		
	**Terbutaline**	**BUD/FORM**	**BUD/FORM vs. terbutaline**	**BUD/FORM vs. formoterol**	**Formoterol vs. terbutaline**	
**Number of patients with episodes with *>* 6 inh./day as needed**						
All patients, n (%)*^†^	278 (24.4)	228 (20.1)	148 (13.4)	0.51 (0.42–0.62)	0.66 (0.53–0.81)	0.77 (0.65–0.92)
				*P* < 0.001	*P* < 0.001	*P* = 0.004
**Exacerbation rate. patient-year**^**-1**^						
All patients, annualised rate (events)*^‡^	0.37 (377)	0.29 (296)	0.19 (194)	0.52 (0.44–0.62)	0.67 (0.56–0.80)	0.78 (0.67–0.91)
				*P* < 0.0001	*P* < 0.0001	*P* = 0.0012
High as-needed group, annualised rate (events)^§^						
*Post-index day to study end*	0.97 (180)	0.76 (120)	0.41 (42)	0.42 (0.29–0.62)	0.54 (0.36–0.80)	0.79 (0.61–1.03)
*≤21 days post-index*	3.04 (48)	2.72 (35)	1.67 (14)	0.55 (0.30–1.00)	0.61 (0.33–1.14)	0.90 (0.58–1.38)
**Severe exacerbation days**						
All patients, % rate (total days)*^║^	0.8 (3030)	0.6 (2214)	0.4 (1353)	0.45 (0.35–0.58)	0.63 (0.48–0.82)	0.72 (0.57–0.91)
				*P* < 0.0001	*P* < 0.001	*P* < 0.001
*Post-index day to study end*	2.4 (1616)	1.7 (998)	0.8 (300)	0.34 (0.21–0.50)	0.46 (0.29–0.71)	0.73 (0.52–1.01)
*≤21 days post-index*	7.9 (458)	6.1 (287)	2.5 (77)	0.32 (0.14–0.57)	0.41 (0.18–0.81)	0.77 (0.44–1.28)
**Hospitalisation/ER visit rate, patient-year**^**–1**^						
All patients, rate (events)^*‡^	0.07 (115)	0.05 (98)	0.04 (70)	0.61 (0.45–0.82)	0.73 (0.54–0.99)	0.83 (0.63–1.08)
				*P* < 0.001	*P* = 0.046	*P* = 0.17
High as-needed group, rate (events)^§^						
*Post-index day to study end*	0.27 (50)	0.24 (37)	0.18 (18)	0.65 (0.38–1.12)	0.75 (0.42–1.31)	0.88 (0.57–1.34)
*≤21 days post-index*	1.14 (18)	1.09 (14)	0.96 (8)	0.84 (0.36–1.93)	0.88 (0.37–2.09)	0.95 (0.47–1.92)

^*^All patients (ITT population); ^†^Cox proportional hazard model (post hoc analysis); ^‡^Poisson regression (a priori analysis); ^§^Poisson regression (*post hoc* analysis); ^║^Bootstrap procedure (*post-hoc* analysis).

BUD/FORM = budesonide/formoterol; CI = confidence interval; ER = emergency room; inh. = inhalations.

**Table 3:** Annualised exacerbation rates and hospitalisations/ER treatments in the full ITT population and the subgroups with episodes of high as-needed reliever use (>6 inhalations/day on ≥1 study day) (Study B)

	**Treatment groups**			**Risk or rate ratios (95% CI)**		
	**SAL/FLU FD + terbutaline**	**BUD/FORM FD + terbutaline**	**BUD/FORM maintenance and reliever therapy**	**BUD/FORM maintenance and reliever therapy vs. SAL/FLU FD + terbutaline**	**BUD/FORM maintenance and reliever therapy vs. BUD/FORM FD + terbutaline**	**BUD/FORM FD + terbutaline vs. SAL/FLU FD + terbutaline**
**Number of patients with episodes with *>* 6 inh./day as needed**						
All patients, n (%)^*†^	156 (13.9)	167 (15.1)	124 (11.2)	0.74 (0.59–0.94)	0.65 (0.52–0.83)	1.13 (0.91–1.41)
				*P* = 0.014	*P* < 0.001	*P* = 0.26
**Exacerbation rate. patient-year**^**-1**^						
All patients, annualised rate (events)*^‡^	0.38 (208)	0.32 (173)	0.24 (125)	0.61 (0.49–0.76)	0.72 (0.57–0.90)	0.85 (0.69–1.04)
				*P* < 0.001	*P* = 0.0048	*P* = 0.10
High as-needed group, annualised rate (events)^§^						
*Post-index day to study end*	1.92 (94)	1.50 (80)	0.92 (37)	0.48 (0.31–0.75)	0.61 (0.39–0.97)	0.78 (0.55–1.11)
*≤21 days post-index*	4.78 (41)	3.02 (28)	2.00 (14)	0.42 (0.23–0.77)	0.66 (0.35–1.25)	0.63 (0.39–1.02)
**Severe exacerbation days**						
All patients, % rate (total days)*^║^	0.7 (1327)	0.6 (1143)	0.4 (692)	0.53 (0.38–0.73)	0.60 (0.42–0.86)	0.88 (0.64–1.21)
				*P* < 0.001	*P* < 0.01	*NS*
High as-needed group, % rate (total days)^║^						
*Post-index day to study end*	3.7 (667)	2.8 (539)	1.8 (259)	0.47 (0.27–0.78)	0.64 (0.34–1.15)	0.74 (0.44–1.21)
*≤21 days post-index*	10.1 (315)	6.0 (203)	3.4 (88)	0.34 (0.14–0.66)	0.57 (0.23–1.16)	0.60 (0.35–1.00)
**Hospitalisation/ER visit rate, patient-year**^**–1**^						
All patients, rate (events)^*‡^	0.16 (106)	0.10 (72)	0.10 (64)	0.61 (0.44–0.83)	0.88 (0.63–1.24)	0.68 (0.51–0.92)
				*P* = 0.0015	*P* = 0.47	*P* = 0.013
High as-needed group, rate (events) ^§^						
*Post-index day to study end*	0.96 (47)	0.74 (40)	0.40 (16)	0.41 (0.23–0.73)	0.53 (0.30–0.95)	0.78 (0.51–1.19)
*≤21 days post-index*	2.44 (21)	1.08 (10)	0.86 (6)	0.35 (0.14–0.86)	0.79 (0.29–2.18)	0.44 (0.21–0.94)

^*^All patients (ITT population); ^†^Cox proportional hazard model (post hoc analysis); ^‡^Poisson regression (a priori analysis); ^§^Poisson regression (*post hoc* analysis); ^║^Bootstrap procedure (*post hoc* analysis).

BUD/FORM = budesonide/formoterol; CI = confidence interval; ER = emergency room; FD = fixed-dose maintenance; inh. = inhalations; SAL/FLU = salmeterol/fluticasone.

### Exacerbation conversion rate from episodes of >6 inhalations/day of reliever

**Figure 3:**
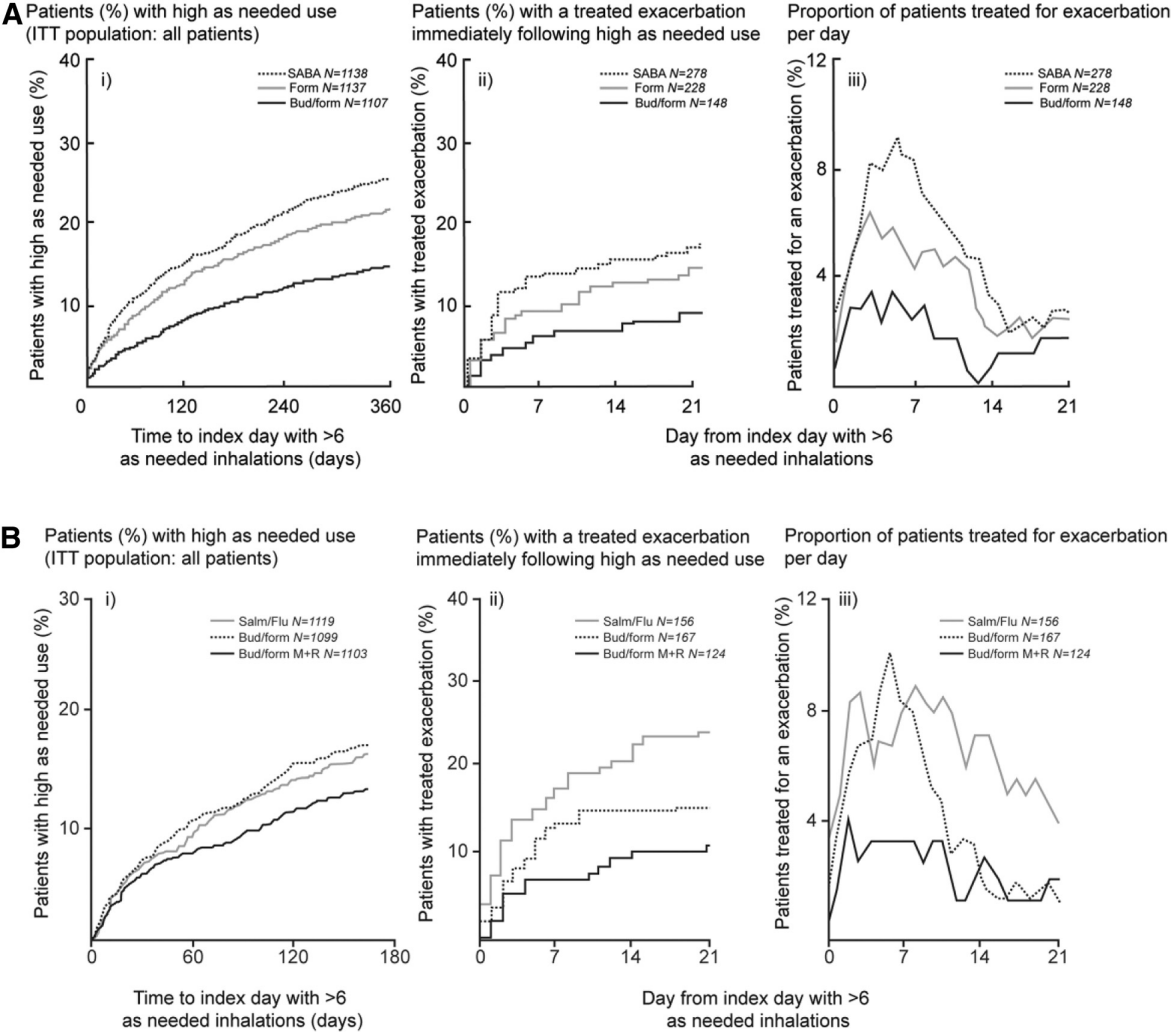
**Asthma exacerbations associated with episodes of high reliever use (>6 inhalations/day) A) in Study A, B) in Study B.** i) Proportion of all patients (ITT population) with an index day of >6 inhalations/day in the three treatment arms over the study period; ii) Kaplan–Meier plot of time from first use of >6 as-needed inhalations/day to first exacerbation during the following 21 days; iii) percentage of patients with ongoing treatment for an exacerbation for each day following the index day. Note in panels ii) and iii) only a minority of the at risk subgroup of patients having an episode of high reliever use identified in panel i) developed a severe exacerbation requiring additional treatment. The majority of high reliever episodes resolved spontaneously on all regimens.4

In the Study A ITT population, 180/377 (48%) exacerbations in the as-needed terbutaline group and 120/296 (41%) in the as-needed formoterol group occurred post-index day (Table 2). The corresponding figure for as-needed budesonide/formoterol was 42/194 (22%). Similar results were seen in Study B, where 94/208 (45%) and 80/173 (46%) exacerbations occurred post-index day in the fixed-dose salmeterol/fluticasone and budesonide/formoterol groups, respectively, with terbutaline used as reliever therapy. In contrast, 37/125 (30%) exacerbations in the budesonide/formoterol maintenance and reliever therapy group occurred post-index day (Table 3). Figure 3 shows the time to first exacerbation during the 21 days post-index with each reliever regimen. The overall exacerbation rates from the index day to study end are presented in Tables 2 and 3.

### Exacerbations rates in the window of opportunity

The rate of severe exacerbations per patient during the 21-day post-index period was increased at least eightfold in all groups from the mean ITT rates in the full dataset. Rates increased from 0.37 to 3.04 per patient-year with terbutaline, 0.29 to 2.72 with formoterol and 0.19 to 1.67 with budesonide/formoterol in Study A (Table 2), and from 0.38 to 4.78 with fixed-dose salmeterol/fluticasone, 0.32 to 3.02 with fixed-dose budesonide/formoterol and 0.24 to 2.00 per patient-year with budesonide/formoterol maintenance and reliever therapy, in Study B (Table 3). In both studies exacerbation rates favoured budesonide/formoterol maintenance and reliever therapy vs. each fixed-dose regimen using SABA. Nevertheless, reliever use remained a robust marker for deteriorating asthma control irrespective of reliever regimen.

### Exacerbation days in the window of opportunity

In the terbutaline as-needed group (Study A) post-index, the daily profile of the percentage of patients with actively treated exacerbations peaked at ~7 days, waning to a more stable level at ~16 days (Figure 3A). In Study B, the peak incidence of exacerbation treatment in patients using budesonide/formoterol with terbutaline as needed was of similar magnitude and duration as in Study A. In both studies, patients using budesonide/formoterol maintenance and reliever therapy had an attenuated peak (Figures 3A and B). The number and duration of individual events contributing to the mean profiles are available online (Additional file 2: Figure S1 and Additional file 3: Figure S2). The average time in an exacerbation state during the 21-day post-index period was 7.9% of days with as-needed terbutaline, 6.1% with as-needed formoterol and 2.5% of days with as-needed budesonide/formoterol, respectively in Study A (Table 2). In Study B, the corresponding values were 10.1% of days with fixed-dose salmeterol/fluticasone plus as-needed terbutaline, 6.0% with fixed-dose budesonide/formoterol plus as-needed terbutaline and 3.4% of days with budesonide/formoterol maintenance and reliever therapy, respectively (Table 3).

### ICS dose in the window of opportunity

Only a minority of patients reported high reliever use; however, because of the regimen nature, patients receiving budesonide/formoterol maintenance and reliever therapy were exposed to increased ICS doses during the window of opportunity. In Study A, total ICS exposure based on median average beclomethasone dipropionate (BDP) equivalents in the 21-day period was 1,363 μg/day with budesonide/formoterol maintenance and reliever therapy vs. 500 μg/day for both comparator groups. In Study B, the corresponding values were 1,357 μg/day with budesonide/formoterol maintenance and reliever therapy vs. 1,000 μg/day for both fixed-dose control groups. Overall, ICS exposure was not excessive: the median ICS doses for budesonide/formoterol maintenance and reliever therapy for the ITT population were 657 and 642 μg/day BDP equivalents in Study A and B, respectively.

### Hospitalisations/ER visits

In Study A, the need for hospitalisation/ER visits was not significantly different between regimens during the 21-day period. This was in contrast to the ITT population. Nevertheless, hospitalisation/ER visits were less frequent with budesonide/formoterol vs. terbutaline (8 vs. 18 events; Table 2). The risk of hospitalisation/ER visits in Study B during the 21-day period was significantly (P < 0.05) lower for both budesonide/formoterol regimens compared with fixed-dose salmeterol/fluticasone (Table 3). This mirrored previous findings in the ITT population.

### Tolerability

In both studies, the number of patients with SAEs and DAEs was lower in the budesonide/formoterol maintenance and reliever therapy arms vs. comparator arms in patients using >6 inhalations/day of reliever (Table 4). Asthma was the only common cause of SAEs and DAEs by event type; all other events occurred with an incidence ≤1%. In Study A, patients were also discontinued due to pre-specified exacerbation/safety criteria (i.e. ≥3 severe exacerbations during any 3 months, five during the whole study or any severe exacerbation lasting ≥20 days). This resulted in an additional 10, 2 and 1 patient(s) in the terbutaline, formoterol and budesonide/formoterol as needed groups, respectively, being withdrawn from the study (Figure 1). In Study A, two deaths were reported – one in the as-needed terbutaline arm (cardiac arrest) and one in the as-needed formoterol arm (brain neoplasm). No deaths were reported in Study B.

**Table 4:** Safety among the subgroups of patients with episodes of >6 inhalations/day of as-needed therapy in Study A and Study B

	**Study A (12-month assessment)**			**Study B (6-month assessment)**		
	**BUD/FORM + terbutaline (n = 278)**	**BUD/FORM + formoterol (n = 228)**	**BUD/FORM maintenance and reliever therapy (n = 148)**	**SAL/FLU FD + terbutaline (n = 156)**	**BUD/FORM FD + terbutaline (n = 167)**	**BUD/FORM maintenance and reliever therapy (n = 124)**
Deaths, n (%)	1 (<0.5)	1 (<0.5)	0 (0)	0 (0)	0 (0)	0 (0)
Patients with an SAE, n (%)	26 (9)	25 (11)	21 (14)	13 (8)	18 (11)	6 (5)
Asthma-related SAE, n (%)	15 (5)	15 (7)	8 (5)	7 (4)	5 (3)	2 (2)
Patients with a DAE, n (%)	12 (4)	12 (5)	2 (1)	3 (2)	6 (4)	0 (0)
*Asthma-related DAEs, n (%)	6 (2)	9 (4)	1 (1)	3 (2)	4 (2)	0 (0)

*An additional 10, 2 and 1 patients receiving: terbutaline, formoterol and budesonide/formoterol reliever regimen, respectively in Study A were withdrawn due to predefined asthma-related treatment failure/safety criteria.

DAE = adverse events leading to discontinuation from the study; BUD/FORM = budesonide/formoterol; FD = fixed-dose maintenance; SAE = serious adverse event; SAL/FLU = salmeterol/fluticasone.

## Discussion

We have shown that episodes of high reliever use (>6 inhalations/day on at least 1 day) predict an increased risk of near-term and later exacerbations, and also that most of these episodes do not develop into severe exacerbations. Our findings suggest that budesonide/formoterol maintenance and reliever therapy can reduce the incidence of episodes of high reliever use and the severe exacerbations that coincide with many of these events compared with ICS/LABA and reliever regimens at similar or higher maintenance ICS doses.

We examined the exacerbation risk immediately following an episode with high reliever use. Unlike previous studies retrospectively examining the association between SABA use from prescription records and the incidence of severe exacerbations, [7,18,19] this analysis focused on the temporal association between the first day with high reliever use and the onset of exacerbation. All patients used standard maintenance ICS/LABAs. When as-needed reliever use exceeded 6 inhalations on at least 1 day, exacerbation rates in the 21 days post-index increased at least eightfold compared with background rates in the ITT populations for all regimens. This suggests that, irrespective of the level of maintenance ICS/LABA treatment and reliever type, a day with high as-needed use is a robust measure of disease instability.

On a background of budesonide/formoterol maintenance therapy, it has been reported that both formoterol and budesonide contribute to decreased severe exacerbations [12] .We have shown that formoterol is superior to terbutaline and that budesonide/formoterol is superior to both in reducing the risk of a high reliever use episode [12]. Budesonide/formoterol is also superior to both alternative relievers in reducing the conversion rate of these episodes to severe exacerbations in the 21 days post-index day. This suggests that both budesonide and formoterol have airway stabilising effects that explain the preventative effect observed in this study and in the ITT analysis.

Budesonide/formoterol as maintenance and reliever therapy was also compared with budesonide/formoterol at twice the maintenance dose and to salmeterol/fluticasone at a similarly higher ICS dose [10]. Notwithstanding the more intense maintenance treatment, patients using budesonide/formoterol maintenance and reliever therapy were less likely than other groups to have an index high reliever use episode. They were also less likely than those on salmeterol/fluticasone fixed maintenance dose to develop a severe exacerbation. There was no significant difference between budesonide/formoterol maintenance and reliever therapy and fixed-dose budesonide/formoterol in this comparison but there was a significant reduction in the number of severe exacerbation days in the 21-day window. Therefore, compared with budesonide/formoterol maintenance and reliever therapy, all tested comparators were associated with an increased risk of events of concern and a higher risk of conversion to a severe exacerbation.

A criticism of the budesonide/formoterol maintenance and reliever studies was that the maintenance dose of the higher dose comparator arms was not adjusted to allow even higher doses, which would be possible in ordinary clinical care. However, even on the higher fixed-dose regimes, the majority of events resolve without a change in treatment. The institution of an even higher maintenance dose would thus have been unnecessary in the majority of occasions. Studies have not shown any advantage of doubling the dose of maintenance ICS during an asthma worsening [20,21]. However, quadrupling or quintupling ICS maintenance doses is reported as effective, [22,23] but these are based on intensive multi-modality asthma monitoring that may be possible in trials but not in ordinary care. In any case, the dose increases with budesonide/formoterol in the 21 days after the onset of the event are less than a trebling of the regular maintenance dose.

The precise mechanism behind this acute protection is uncertain but may rely on several factors, including reductions in the late asthmatic response after allergen inhalation, eosinophilic inflammation, bronchial hyperreactivity and/or reducing pulmonary blood flow, all of which occur within hours of administering higher ICS doses [24-27].

In the current analysis, patients receiving budesonide/formoterol maintenance treatment plus formoterol as reliever had a lower risk of severe exacerbations compared with a control subgroup using equally high levels of terbutaline. Furthermore, in a smaller group of patients with episodes of high as-needed budesonide/formoterol use, there was a further reduced incidence of exacerbations including asthma-related SAEs and DAEs compared with the larger groups using LABA or SABA as reliever. A meta-analysis of seven studies[9-15] has also confirmed that the incidence of asthma-related SAEs and DAEs with budesonide/formoterol maintenance and reliever therapy is significantly lower than with fixed-dose regimens using ICS or ICS/LABA combination therapy plus terbutaline or salbutamol as needed [28]. Thus, budesonide/formoterol maintenance and reliever therapy appears to be well tolerated and reduces the risk of severe exacerbations following exposure to high levels of SABA that could mask worsening inflammation.

The present analysis also provided an insight to the a priori significant increase in ER visits/hospitalisations with fixed-dose salmeterol/fluticasone compared with both budesonide/formoterol regimens independent of reliever therapy (Table 4) [10]. Patients receiving salmeterol/fluticasone had a longer peak period with increased exacerbation treatment and a significant doubling in ER visits/hospitalisations during the 21 days post-index vs. both budesonide/formoterol regimens (Figure 3C; Table 3). This difference occurred without any increase in the number or duration of episodes of high terbutaline use with fixed-dose salmeterol/fluticasone vs. fixed-dose budesonide/formoterol. Furthermore, there was no difference in asthma control between the fixed-dose subgroups at baseline or on treatment outside of the 21-day window. Thus, the current analyses highlight a difference between fixed-dose ICS/LABAs, not during stable periods, but impacting outcomes during periods of instability. The varying pharmacological properties of the different LABAs, primarily the lower efficacy of salmeterol vs. formoterol leading to less broncho protection, [29,30] antagonism of SABA-induced smooth muscle relaxation [31] and broncho protection [30] with salmeterol but not formoterol, may provide a rationale for the differences in exacerbation rates seen between LABAs when added to ICS in two meta-analyses [32,33] .Our observations may warrant additional prospective investigation.

This analysis does have limitations. The number of high reliever use events can be compared between treatment arms. However, subsequent comparisons may be influenced by the efficacy of randomised treatment and patients included in further analysis may be dissimilar between groups if groups are diminished in size due to differences in the prior efficacy of the regimens. Despite these difficulties it is an important observation that high reliever use episodes are more likely to resolve without severe exacerbation if they occur in a person who is receiving budesonide/formoterol maintenance and reliever therapy than in someone who remains on standard reliever therapy.

In summary, this study confirms that a window of opportunity exists for preventing exacerbations associated with high reliever use. When compared with alternative fixed-dose ICS/LABA plus SABA regimens, at similar or higher maintenance ICS doses, budesonide/formoterol maintenance and reliever therapy reduces the incidence of episodes of high reliever use and exacerbations that frequently coincide with acute periods of asthma instability. Budesonide/formoterol maintenance and reliever therapy is a logical evolution from the fixed-dose maintenance treatment strategy that is more efficient and effective in patients with moderate or severe asthma.

## Abbreviations

FEV, Forced Expiratory Volume; ICS, Inhaled CorticoSteroid; LABA, Long-Acting β2-Agonist; SABA, Short-Acting β2-Agonist.

## Competing interests

RB has received reimbursement for attending scientific conferences, and/or fees for speaking and/or consulting from AstraZeneca, Boehringer Ingelheim, Chiesi, GlaxoSmithKline, Novartis, Nycomed and Pfizer. The Pulmonary Department at Mainz University Hospital received financial compensation for services performed during participation in clinical trials organised by various pharmaceutical companies. PK has received a fee for speaking at a company-sponsored symposium from AstraZeneca and travel to an international congress was funded by AstraZeneca. MJP has received honoraria from AstraZeneca for CME lectures and has acted on advisory boards for AstraZeneca, GlaxoSmithKline and Nycomed. TLGA, IPN and SP are employees of AstraZeneca and hold shares in AstraZeneca. KR has participated in advisory boards and received lecture fees from AstraZeneca, Boehringer, Chiesi Pharmaceuticals, Pfizer, Nycomed, MSD and GlaxoSmithKline. The Department of Pulmonology at Leiden University Medical Centre has received grants from various pharmaceutical companies.

## Author contributions

RB was involved in the study design and the drafting of the manuscript. SP performed the statistical analysis. All authors read and approved the final manuscript.

## Supplementary Material

Additional file 3 Figure S2: P Incidence, type and duration of asthma exacerbations associated with episodes of high reliever use of >6 inhalations/day in Study B. Click here for file

Additional file 1 Table S1: Patient baseline demography in full ITT populations). Click here for file

Additional file 2 Figure S1: Incidence, type and duration of asthma exacerbations associated with episodes of high reliever use of >6 inhalations/day in Study A. Click here for file
