# Supplementary material for: The effect of budesonide/formoterol maintenance and reliever therapy on the risk of severe asthma exacerbations following episodes of high reliever use: an exploratory analysis of two randomised, controlled studies with comparisons to standard therapy
Source: Respir Res. 2012 Jul 20;13(1):59. doi: 10.1186/1465-9921-13-59 (PMC3561645; doi:10.1186/1465-9921-13-59)
Supplement: Additional file 1 — Table S1: Patient baseline demography in full ITT populations). [file 1465-9921-13-59-S1.pdf]

SUPPLEMENTARY TABLE 1: Patient baseline demography in full ITT populations

	Study A			Study B		
	BUD/FORM + terbutaline (n=1,141)	BUD/FORM + formoterol (n=1,140)	BUD/FORM maintenance and reliever therapy (n=1,113)	SAL/FLU FD + terbutaline (n=1,123)	BUD/FORM FD + terbutaline (n=1,105)	BUD/FORM maintenance and reliever therapy (n=1,107)
Male, n (%)	450 (39)	458 (40)	437 (39)	484 (43)	448 (41)	479 (43)
Age, years	43 (12–83)	42 (12–81)	42 (12–89)	38 (12–83)	38 (12–83)	38 (11–79)
ICS at entry, $\mu g \cdot day^{-1}$	751 (250–1,600)	758 (320–1,600)	757 (160–1,600)	744 (200–2,000)	750 (100–3,200)	740 (250–2,000)
FEV_1, % predicted	72 (39–100)	72 (38–115)	72 (30–110)	73 (30–143)	73 (46–122)	72 (29–131)
Reversibility, %	24 (11–90)	24 (0–96)	24 (6–132)	23 (3–92)	25 (7–150)	24 (10–106)
Rescue inhalations/day	1.9 (0.3–9.7)	1.9 (0–9.1)	1.8 (0–8.9)	2.3 (0–10.8)	2.3 (0.2–9.5)	2.3 (0–12.6)

Data are presented as mean (range) unless otherwise stated. In Study A all groups received budesonide/formoterol 160/4.5 µg bid during the run-in and in all groups in the randomised treatment period. In Study B patients were treated with maintenance ICS alone at the level used at study entry during run-in. BUD/FORM = budesonide/formoterol; SAL/FLU = salmeterol/fluticasone 50/250 µg bid; FD = fixed-dose maintenance; FEV_1 = forced expiratory volume in 1 second; ICS = inhaled corticosteroids; LABA = long-acting β_2-agonist
