# Supplementary material for: The effect of budesonide/formoterol maintenance and reliever therapy on the risk of severe asthma exacerbations following episodes of high reliever use: an exploratory analysis of two randomised, controlled studies with comparisons to standard therapy
Source: Respir Res. 2012 Jul 20;13(1):59. doi: 10.1186/1465-9921-13-59 (PMC3561645; doi:10.1186/1465-9921-13-59)
Supplement: Additional file 2 — Figure S1: Incidence, type and duration of asthma exacerbations associated with episodes of high reliever use of >6 inhalations/day in Study A. [file 1465-9921-13-59-S2.pdf]

SUPPLEMENTARY FIGURE 1: Incidence, type and duration of asthma exacerbations associated with episodes of high reliever use of >6 inhalations/day in Study A

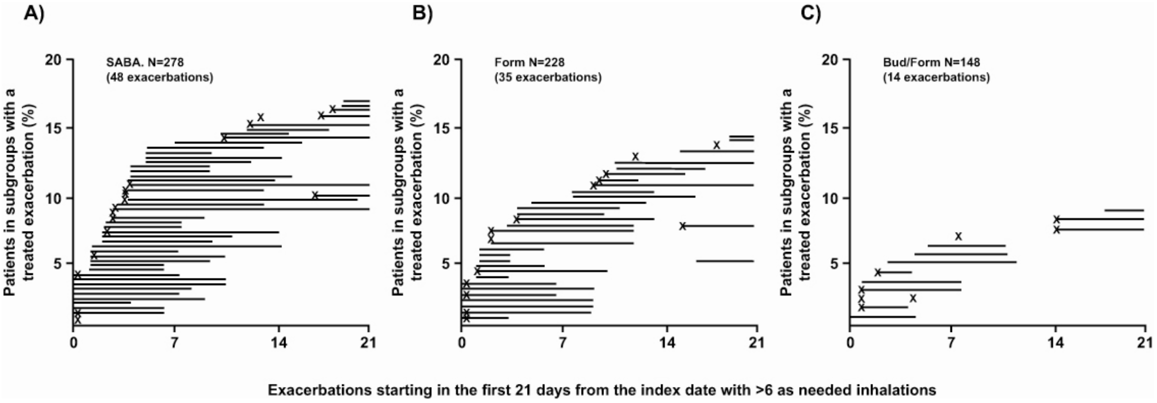

A–C) Incidence of exacerbations including the duration of each event and the type of clinical intervention needed; events marked by an 'X' indicate exacerbations resulting in hospitalisation/ER visits. Only the first 21 days are shown following the index day.
